# Local preferences for three indigenous oil-seed plants and attitudes towards their conservation in the Kénédougou province of Burkina Faso, West-Africa

**DOI:** 10.1186/s13002-020-00393-1

**Published:** 2020-07-23

**Authors:** Fanta Reine Sheirita Tiétiambou, Kolawolé Valère Salako, Jésukpégo Roméo Tohoun, Amadé Ouédraogo

**Affiliations:** 1 Centre Universitaire de Gaoua, Université Nazi BONI, 01 BP 1091, Bobo-Dioulasso, 01 Burkina Faso; 2 Laboratoire de Biologie et Ecologie Végétales, Université Joseph KI-ZERBO, 03 BP 7021, Ouagadougou, 03 Burkina Faso; 3 Laboratoire de Biomathématiques et d’Estimations Forestières, Faculté des Sciences Agronomiques, Université d’Abomey-Calavi, 04 BP, 1525 Cotonou, Bénin; 4 Evolution Biologique et Ecologie, Faculté des Sciences, Université Libre de Bruxelles, CP160/12, Av. F. D. Roosevelt 50, BE-1050 Bruxelles, Belgique

**Keywords:** Conservation actions, Local knowledge, Kénédougou, Plus-tree, Underutilized-plants, Use value

## Abstract

**Background:**

*Carapa procera*, *Lophira lanceolata*, and *Pentadesma butyracea* are three underutilized but increasingly threatened indigenous oil-seed tree species (IOS) in tropical Africa. Because local knowledge is vital for sustainable management, this study investigated the socio-economic factors that explain local people’s (i) preferences for these IOS, (ii) attitudes toward their conservation, and (iii) ability to identify “plus trees” based on seed traits. We predicted a positive relationship between response variables and informants’ age, residence status, gender (femaleness), and existence of market opportunities for each IOS. We also predicted that a higher preference for a given IOS has a positive effect on people’s attitudes for its conservation and the aptitude to identify its “plus trees.” We additionally expected significant differences among ethnic groups for each response variable.

**Methods:**

Data were collected through individual semi-structured interviews with 336 informants from 14 randomly selected villages in the species distribution area of Kénédougou province. For each species, the collected data were the number of actual uses reported (converted to use value—UV, as a measure of the species preference), practiced conservation actions (converted to conservation attitude using a four-scale scoring method), and possible criteria for selecting preferred trees for seed oil extraction. Generalized linear mixed models were used to test for the fixed effects of socio-economic factors, and account for the random variation across villages.

**Results:**

The results showed species-specific patterns. *Carapa procera* had the highest UV and hence was the most preferred IOS, particularly by women. Informants from the Siamou ethnic group had the highest UV irrespective of IOS. The most cited conservation actions were assisted natural regeneration and banning of tree cutting, which were practiced for *C. procera* and *L. lanceolata*. No conservation measure was cited for *P. butyracea*. The practice of tree planting was not recorded for any of the IOS. Young and male informants participated less in conservation actions. Tree selection for oil-seed collection was mainly guided not by “oil extraction yield” but rather by the “quality of extracted oil” (namely oil color and taste for food uses, and oil bitterness for medicinal efficacy). The selection mainly concerned *L. lanceolata* and was mostly practiced by elderly people.

**Conclusion:**

This study provided useful local knowledge-based information to guide conservation actions and valorization strategies of three IOS. The study sheds further light on the socio-economic factors that are associated to local people’s preferences, conservation attitudes, and individual tree selection.

## Background

Rural communities substantially rely on natural lands and resources for their livelihoods. They subsequently have good knowledge of their environmental resources [1, 2]. This knowledge evolves through a dynamic process of knowledge acquisition and loss to adapt to changing living conditions and needs [3]. Increasingly, such knowledge has proven vital for conservation but also for the domestication of wild tree species with a high potential for being promoted. Some of these species are still underutilized and are threatened by several factors (e.g., overexploitation, habitat fragmentation, climate change, and invasive species). This is particularly the case for *Carapa procera* DC., *Lophira lanceolata*, Tiegh. ex Keay, and *Pentadesma butyracea* Sabine, three multipurpose indigenous oil-seed tree species in Western Burkina Faso [4, 5].

*Carapa procera*, locally called “Kobiyiri” in Djula (a common local language in the Western provinces of Burkina Faso), is a tree of 8–20 m height that naturally occurs in gallery forests of semi-arid regions [6]. The annual seed production per tree varies between 0.7 kg and 30.1 kg of dry material with an annual potential productivity of 1.02 t.ha^−1^ [4]. Seed oil of this species is used as a component for human medicine, cosmetics, and biopesticides [5, 7]. The seed oil is sold on local markets in West Africa and the price varies between €1.5 and €7.7 per liter [4]. *Lophira lanceolata*, called “Mananyiri” in Djula, is a rather small tree of 8–10 m height occurring in the Sudano-Guinean and Guinean savannahs of Africa [6, 8]. In Cameroon, a liter of seed oil from this species fluctuates between €1.8 and €2.8 on the local market [9]. *Pentadesma butyracea*, called “N’taman” in Djula, is a tree species of up to 10 m height and, like *C. procera*, it naturally occurs in gallery forests of semi-arid regions [6]. Its annual seed production varies between 0.7 kg and 20 kg of dry material per tree with an annual potential productivity of 0.36 t.ha^−1^ [4]. Its seed oil is similar to that of shea butter and commonly used for human food and in cosmetics. In some West-African markets, 1 L of its oil is sold at €2–8 [10]. All three species are multi-purpose trees with a high potential for seed oil extraction. Seeds of these species have a high content of oil, the therapeutic and cosmetic virtues of which are well documented in many African countries [5, 7, 10–12]. In Kénédougou, different parts of these plants are used by rural communities in cosmetics for body and hair care, pharmacopeia for health care, handcrafts for construction, and biopesticides for phytosanitary treatments [7, 13, 14].

During recent decades, repeated and longer droughts in addition to habitats fragmentation due to extensive agriculture have put high pressures on tree species across the Sahel [15, 16]. Overharvesting of fruits and seeds makes their natural stands vulnerable to aging because of the threat on natural regeneration [2, 14, 17, 18]. This is particularly true for *C. procera*, *L. lanceolata*, and *P. butyracea* the seeds of which provide high use value oils. Therefore, actions for conservation and cultivation of these species have become urgent to guarantee the current and future optional sustainable uses of their products.

In most developing countries, there is a global deficiency of government-driven policies to support the valorization, conservation, and cultivation of wild species. In Burkina Faso, most of the efforts were on exotic species (e.g., *Anacardium occidentale* L., *Azadirachta indica* A. Juss., *Eucalyptus camaldulensis* Dehn., and *Mangifera indica* L.) which were used in several tree planting programs as a strategy to mitigate the impacts of climate variability and change on farmers’ food security and livelihoods [19]. Nonetheless, farmers themselves have developed some traditional conservation practices such as farmers’ management of natural regeneration. They have even initiated cultivation (i.e., tree planting) of some indigenous species (e.g., *Adansonia digitata*, *Vitellaria paradoxa*, *Ziziphus mauritiana*) [19–22], the understanding of which provides important baseline information for further actions [23–25]. For example, in the Siamou ethnic group of Burkina-Faso in Kénédougou province, women raise seedlings of *C. procera* in nurseries and sell them to local people for plantation. Attitudes toward the conservation of species are, however, species-specific [26] and depend on several factors including the socio-demographic profiles (gender, age, education, ethnicity, etc.) of local people [19, 27], their geographical location and preferences (local importance of species: UV, market value) [28], and their knowledge on the species biology [29, 30]. Traditional conservation and management actions are diverse and may range from plantations where a species is deliberately planted, to assisted natural regeneration (ANR). In ANR, seedlings and saplings of targeted species are protected and maintained for their survival and development; individuals of the species are not subject to logging [31]. Other species may not be the subject of any specific conservation and cultivation practices even though their local importance is recognized [31].

Indigenous oil-seed species (IOS) can display large morphological variations in their fruits, seeds, kernels, oil, etc. [8, 32, 33], which results in interesting material variants (i.e., morphotypes or so-called plus trees) that are perceived and valued by local people. Therefore, individual trees presenting interesting characteristics might be particularly targeted for seed collection not only for consumption and other uses but also for conservation and possibly cultivation. It is subsequently expected that local people would have some local selection criteria of those materials [34]. However, there is a risk of genetic erosion due to underutilization or unsuitable management of the material perceived as “non-interesting” [34]. Species subject to such selections deserve particular conservation actions to guarantee the conservation of genetic resources. Understanding local people’s ability to select plant materials, concerning their uses and conservation, therefore, has evident implications for the management of the genetic resources of the concerned species. It is expected that species with high use and market values will likely be subject to such selection as long as a morphological diversity is perceived and has implications for the quality of derived products.

**Fig. 1:**
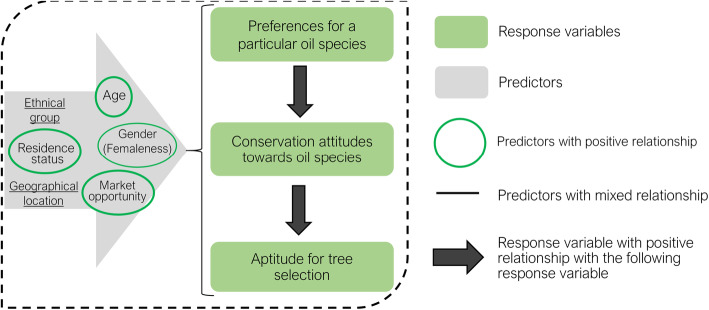
Model of multiple hypotheses regarding local preferences of species, local people conservation attitude toward the species, and local people’s aptitude for selection of plus tree. All variables in the gray arrow, namely, gender, age, residence status, ethnic group, market opportunity, and geographical location (villages) are explanatory variables for all three response variables. Gender had two levels: men (coded 1) and women (coded 0). Residence status had two levels: indigenous (coded 1) and non-residents (coded 0). Existence of market opportunities also had two levels: Yes (coded 1) and No (coded 0). Details on response variables can be found in the section data processing and analysis

In this study, we aimed to contribute to a better management of the three IOS by understanding local people’s (i) preferences, (ii) attitudes toward conservation, and (iii) ability to identify the plus trees of *C. procera*, *L. lanceolata*, and *P. butyracea* in the Kénédougou province, Burkina Faso. The study was driven by a model of multiple hypotheses inspired by Whitney et al. [35] in regards to preferences for species, conservation of species, and selection of plus trees, and which are summarized in Fig. 1.

Theories suggest that an individual’s socio-cultural and demographic characteristics such as gender, age, and ethnicity influence their preference or UV of a given species [27]. In particular, because women are specialized in the collection of non-timber forest products [36, 37] such as IOS (e.g., the shea butter tree *Vitellaria paradoxa* [38]), they are likely to have greater knowledge and UV of IOS. Similarly, knowledge accumulation is a time-dependent process, and older people are expected to have greater knowledge and hence utilize a species more than younger people [39]. Furthermore, due to historical differences in habits and customs, individuals belonging to different ethnic groups are likely to have different knowledge about the uses of a given species, even in the same geographical area [40, 41]. Also, individuals belonging to the same ethnic group but living in a different geographical area can have different knowledge and values about a given species because other species better fulfill the role of that plant in different geographical locations [42]. Compared to non-residents, resident individuals might have more knowledge and value for a given species [5, 43]. For example, Cuni-Sanchez et al. [44] reported that pastoralists identified fewer ecosystem services than resident farmers, used some ecosystem services differently, and had limited interest in forest conservation. Proximity to market opportunities for local resources has a positive impact on how local people value and use a given species [45]. In particular, species with market opportunities either local or regional are more inclined to be collected, used, and maintained than species with no market opportunities [46]. Moreover, people’s preferences for a given species determine their conservation attitudes toward that species [26], such that species with a higher preference are expected to benefit from better conservation attitudes. Finally, people having higher preferences for a given species are likely to have a better knowledge of interesting material within that species and possibly select individuals with the most interesting traits for use, cultivation, and markets. Based on the above, and as illustrated in Fig. 1, we predicted a positive relationship between informants’ age, residence status, gender, and existence of market opportunities, and each of our variables of interest namely preferences, conservation attitude, and ability to select plus trees of each IOS. We also predicted that a higher preference for a given IOS induces better conservation attitudes and a higher aptitude to identify plus trees of the IOS. We finally expected significant differences among ethnic groups regarding preferences, conservation attitude, and ability to select plus trees of each IOS; mainly because differences in habits, customs, and lifestyles which are inherent to ethnic groups, are likely to affect their perception, uses, valuation, and management of resources in their environment [44].

## Methods

### Study area

**Fig. 2:**
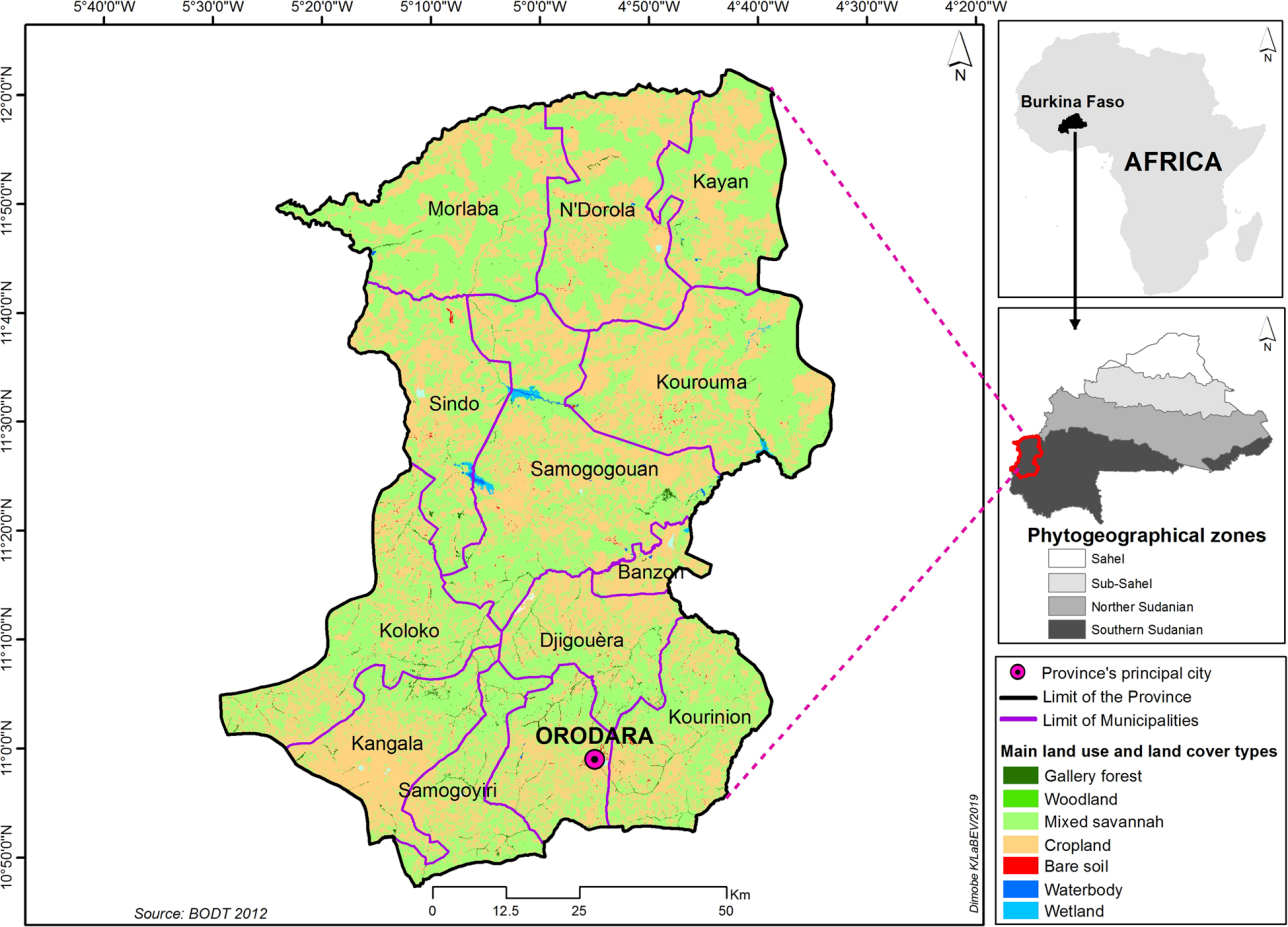
Geographical location of the study area

The study was carried out in Kénédougou province, located in the West of Burkina Faso in the south-Sudanian phytogeographic zone with a Sudanian climate (Fig. 2) [47]. This province covers 8403 km^2^, with 13 administrative communes including an urban one, Orodara, and 176 villages [43]. Kénédougou province is located in the rainiest part of the country and is an area of several humid savannahs and gallery forests, which are the habitats of the three species. From 1983 to 2014, the mean annual rainfall was 1008 ± 164.7 mm, and the annual temperature ranged from 25 °C to 31 °C. Inhabitants were estimated to be 334,751 people in 2011, shared among the Toussian, Bolon, Siamou, Fulani, and Sénoufo ethnic groups. The socio-economic activities are mainly agriculture, livestock breeding, and non-timber forest product collection.

### Sampling design and data collection

Fourteen villages were selected from the distribution range of the three species using a random sampling scheme. For the selected villages, selection of informants was stratified, based on the four major ethnic groups in the province (i.e., Bolon, Siamou, Toussian, and Fulani). Sénoufou ethnic group was not considered because they were already part of a previous study [5]. Among these communities, Fulani are nomadic (non-residents) and the other three groups are indigenous. Eighty-four informants equally distributed between men and women and grouped into three age categories (*young*: < 20 years old, *adults*: 20–50 years old, and *old persons*: > 50 years) [43] were randomly selected from each ethnic group, making a total sample size of *n* = 336 interviewees. Individual semi-structured interviews were conducted using a questionnaire to collect data from informants about which of the IOS, they knew about, the different uses they knew and practiced for each species, and the used plant parts. Questions were also related to the informant’s actions toward the conservation of each species. The responses could be “no action of conservation,” “banning tree cutting,” “assisted natural regeneration,” or “tree planting” following Balima et al. [31]. Finally, informants were asked about their ability to recognize particular tree morphotypes (i.e., plus trees) with regards to their interesting traits for seed oil production, and if so, the criteria they used for such selection. Informants were also asked whether market opportunities exist for each of the three IOS products in their village.

### Data processing and analysis

First, a preference for IOS by rural communities was captured through the calculation of the actual UV index (mean of the number of distinct actual uses reported per informant). It was assumed that a group with high UV for a species prefers that species more than a group with low UV; similarly, a species with high UV is more preferred than a species with low UV [48]. UV is a measure of species relative importance that combines species versatility (the number of distinct uses of a species) and popularity (the number of people who recognize a species as being useful) [49, 50]. Thus, the preferences of informants for each species were measured based on their actual UV. We distinguished between theoretical (practiced and not practiced uses) and actual (practiced) uses, hence, theoretical and actual UV. We further examined for each species the correlation between theoretical UV and actual UV. Next, a four-scale scoring system was used for the attitudes toward IOS conservation as follow: “no action of conservation” (score = 0); “banning tree cutting” (score = 1), “assisted natural regeneration” (score = 2), and “planting trees” (score = 3) (hence, ordinal data). Finally, the aptitude for tree selection was considered as a binary response variable (Yes = 1, No = 0). A Poisson generalized linear mixed model [51], an ordinal logistic mixed model [52], and binomial logistic mixed model [51] were used to examine the effect of explanatory factors on the preference, attitudes toward conservation, and ability to identify plus trees, respectively, based on the multiple hypotheses diagram in Fig. 1. All factors in Fig. 1 were considered fixed, and the village was included as a random factor because the studied villages were selected randomly. This random effect was used as a measure of variation across geographical locations, after controlling for all other sources of variation. The model containing all explanatory variables and the random factor was first established. Then, the parsimonious model was selected using backward elimination based on likelihood ratio tests. By including the village as a random factor in the model, it was assumed that the fluctuation around the intercept, for each village, was normally distributed with a certain variance. Thus, the higher the variance, the greater the differences among villages. The marginal and the conditional *R*^2^ were extracted to compare the effect of the random factor to the fixed ones. Comparable values of marginal and conditional *R*^2^ indicated that most of the variation explained in the models was due to fixed factors, rather than by village random effects. All statistical analyses were carried out with the R statistical software 3.3.0 [53]. The ordered logistic regression models were performed using the “ordinal” package [54]. The mixed Poisson and binomial logistic GLMM were performed using the “lme4” package [55].

## Results

### Factors explaining the actual UV of IOS

**Fig. 3:**
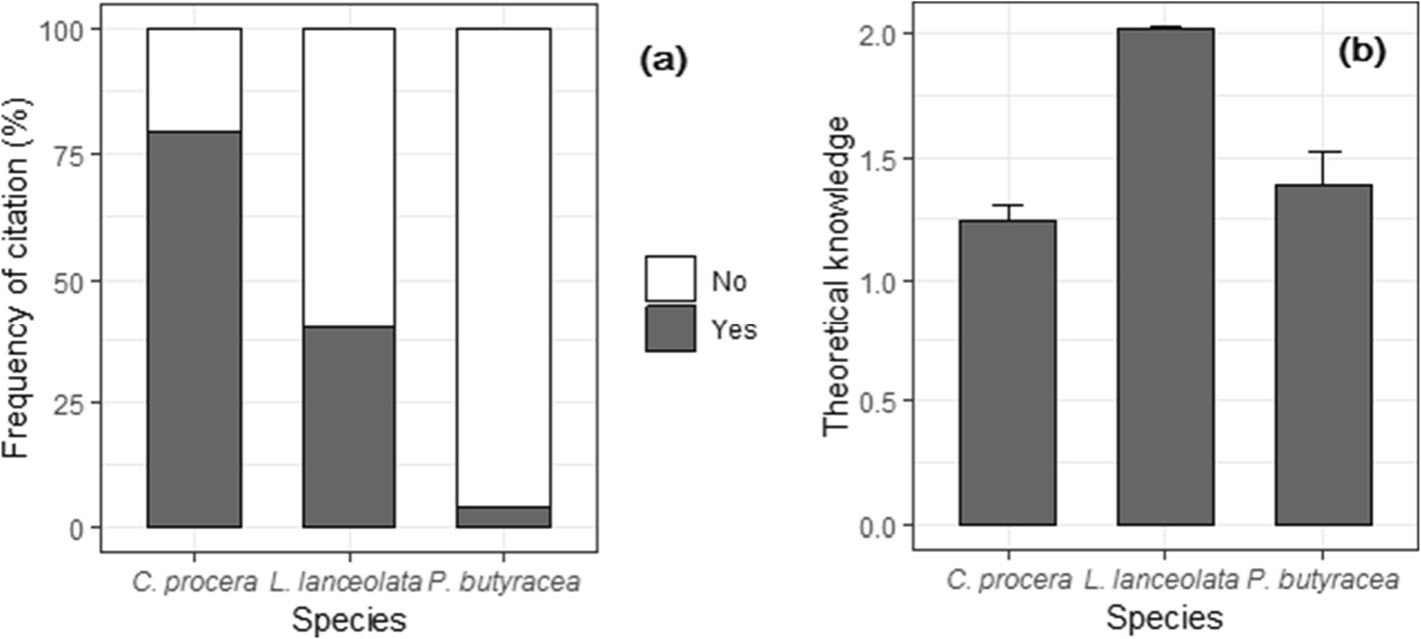
Proportion of informants who knew the three IOS (**a**), and traditional theoretical knowledge of their uses (**b**)

*Carapa procera* was the most known species (79% of informants, Fig. 3a) whereas *P. butyracea* was the least known (3%). However, theoretical knowledge on the uses of *L. lanceolata* was higher than that of *C. procera* and *P. butyracea* (Fig. 3b). The actual UV was significantly (*p* = 0.002, Poisson GLM) higher for *C. procera* (0.61 ± 0.06) and *L. lanceolata* (0.44 ± 0.03), the UV of which were > 15 and 11 times higher, respectively, than that of *P. butyracea* (0.04 ± 0.01). There were also positive and significant (*p* = 0.001) correlations between theoretical and actual UV for each IOS: 0.43, 0.85 and 1.00 for *C. procera*, *L. lanceolata*, and *P. butyracea*, respectively.

**Fig. 4:**
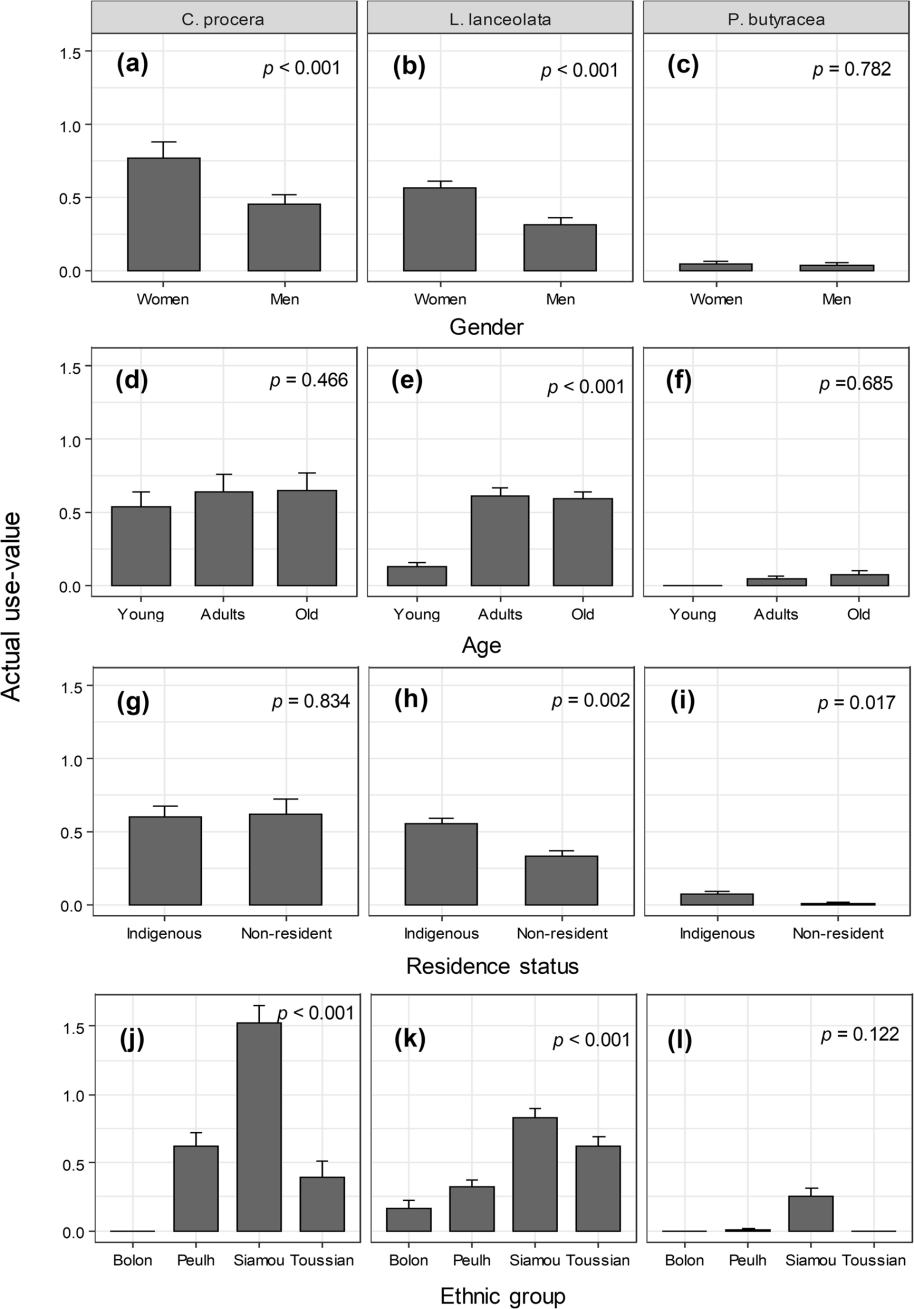
Relationship between actual UV of IOS and informants’ gender, age, ethnic group, and residence status

Univariate models indicated significant effects of gender and ethnic group on UV of *C. procera*; gender, age, residence status, and ethnic group on UV of *L. lanceolate*; and residence status on UV of *P. butyracea* (Fig. 4). The parsimonious model from the model selection procedure indicated that socio-demographic factors associated with the actual UV were species-specific (Table 1). Only gender was significantly associated with the actual UV of *C. procera*, whereas informant age and ethnic group in addition to gender were significantly associated with the actual UV of *L. lanceolata* (Table 1). Only residence status was associated with the actual UV of *P. butyracea* (Table 1). Where a gender effect was significant, men had lower actual UV (Table 1 and Fig. 4a, b, c). Where age exhibited a significant effect, younger informants had lower actual UV compared to adult and old informants, who had similar actual UV (Table 1 and Fig. 4d, e, f). Where residence status had a significant effect, indigenous informants had higher actual UV (Table 1 and Fig. 4g, h, i). Concerning ethnic groups, informants from the Siamou ethnic group had the highest UV whereas Bolon had the lowest UV (Table 1 and Fig. 4j, k, l). Values of conditional and marginal *R*^2^ showed that contrary to *L. lanceolata* (comparable values) for which the random effect of the village was negligible, for the other two species, there was greater variation among villages (large differences between both *R*^2^) with respect to the actual UV (Table 1), reflecting the effect of geographical location.

**Table 1:** Socio-demographic factors associated with actual use-value UV of the three IOS: summary of the parsimonious Poisson generalized mixed model

Terms of the model	*C. procera*			*L. lanceolata*			*P. butyracea*		
	est. (se)	*Z*	*p.*	est. (se)	*Z*	*p.*	est. (se)	Z	*p.*
Intercept	−9.49 (2.53)	−3.75	0.001	−1.22 (0.37)	−3.30	0.001	−12.06 (3.34)	−3.60	0.001
Age (*years old*)	-	-	-	−0.03 (0.17)	−0.17	0.863	-	-	-
*Young*	-	-	-	−1.58 (0.29)	−5.38	0.001	-	-	-
Gender (*men*)	−0.53 (0.14)	−3.66	0.001	−0.58 (0.17)	−3.40	0.001	-	-	-
Residence status (*indigenous*)	-	-	-	-	-	-	2.48 (1.04)	2.39	0.017
Ethnic group (*Fulani*)	-	-	-	0.67 (0.38)	1.78	0.074	-	-	-
*Siamou*	-	-	-	1.60 (0.39)	4.16	0.001	-	-	-
*Toussiam*	-	-	-	1.32 (0.38)	3.44	0.001	-	-	-
Var _Village_	88.38			0.00			60.22		
R^2^_Marginal_	0.07			42.65			2.13		
R^2^_Conditionnel_	90.07			42.65			85.10		

Reference levels were *Women* for gender, *Adult* for age, *Non-resident* for residence status, and *Bolon* for ethnic group

*est.* estimates, *se* standard error, *Z Z* statistics, *p. p* value

Only terms of significant factors are shown, - = non-significant terms

### Factors affecting local people’s attitudes towards IOS conservation

#### Overall pattern of conservation practices

**Fig. 5:**
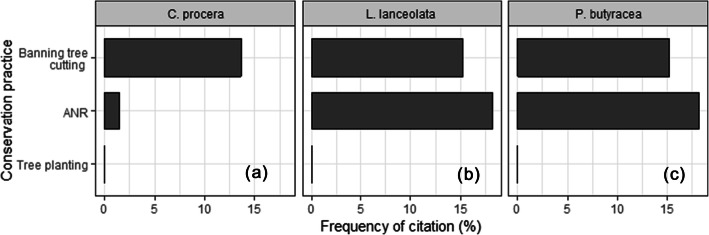
Proportion of people citing conservation practices. ANR, assisting natural regeneration

No conservation measure was recorded for *P. butyracea* in Kénédougou province. Tree planting was not recorded for any species (Fig. 5). Only ANR and banning of tree cutting were practiced for *C. procera* and *L. lanceolata*. People were relatively similarly engaged in ANR for *L. lanceolata* (15%) and *C. procera* (13%). However, they were more inclined to ban tree cutting for *L. lanceolata* (18%) than for *C. procera* (only 3%). Overall, whereas no conservation practice was recorded for *P. butyracea*, *L. lanceolata* received relatively better conservation attitudes than *C. procera* (Fig. 5).

#### Factors affecting conservation attitudes towards IOS

**Fig. 6:**
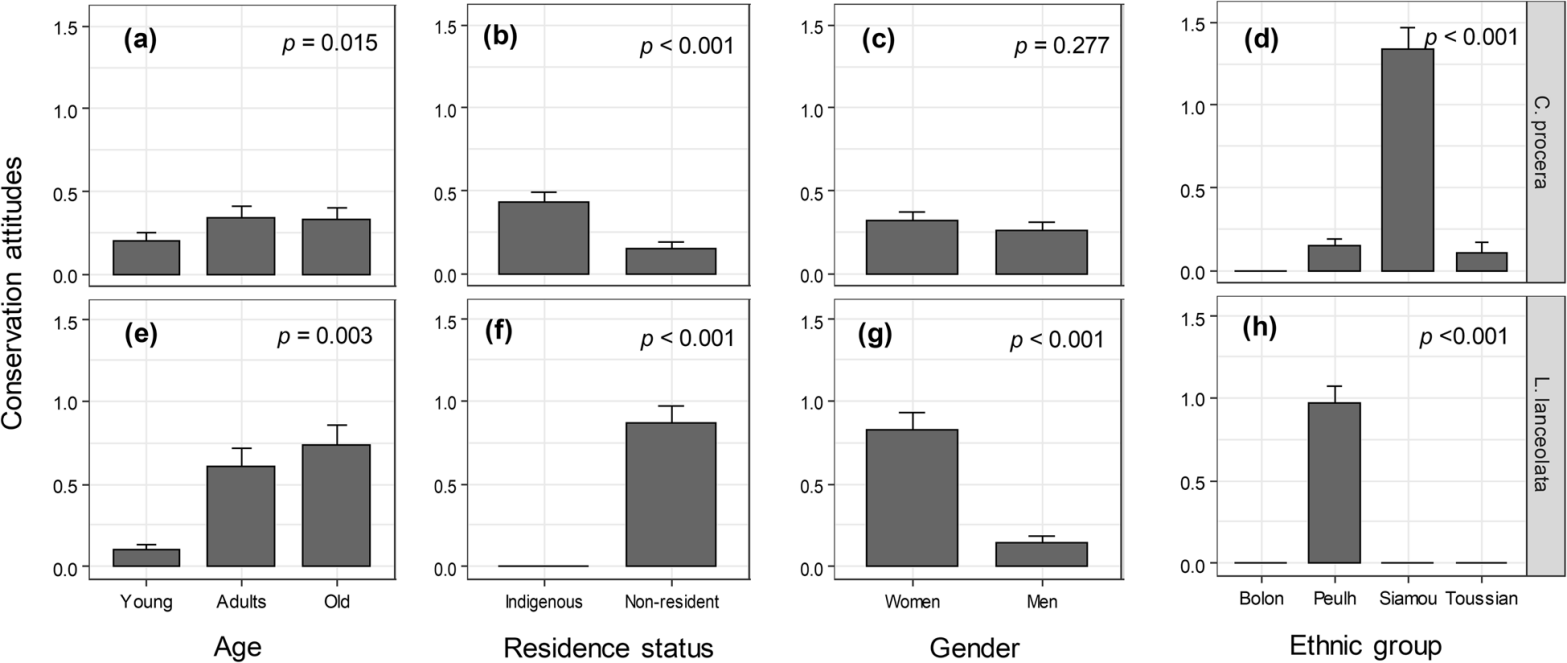
Relationships between conservation attitude and informants’ gender, age, ethnic group, and residence status

This analysis was done only for *C. procera* and *L. lanceolata* because no conservation measure was recorded for *P. butyracea*. Univariate models indicated significant effects of age, residence status, and ethnic group on the score of conservation attitude for *C. procera*, whereas age, residence status, gender, and ethnic group had a significant effect on the score of conservation attitude for *L. lanceolata* (Fig. 6). The parsimonious model from the model selection procedure indicated that the socio-economic factors associated with conservation attitudes toward *C. procera* and *L. lanceolata* were not the same (Table 2). Age of informants, residence status, and oil market opportunities were identified as significant factors influencing conservation attitude toward *C. procera*. Adult and old informants showed similar and better conservation attitudes for *C. procera* than young informants (est._Young_ = −1.90, *p* = 0.006). Informants in villages with market opportunities for *C. procera* oil showed better conservation attitudes (est. = 5.35, *p* = 0.027) than informants in villages with no market opportunities (Table 2). Similarly, indigenous informants had better conservation attitudes (est._Indigenous_ = 2.90, *p* = 0.001) for *C. procera* than non-residents. Concerning *L. lanceolata*, only age and gender were significantly associated with conservation attitudes for this species. As for *C. procera*, adult and older informants had a better conservation attitude for *L. lanceolata* than younger informants. Women had better conservation attitudes (est._Men_ = −1.45, *p* = 0.001) than men towards *L. lanceolata*. The among-village variation was less important for *L. lanceolate*. This was in contrast to *C. procera*, which showed a significant large variation across villages (see differences between conditional and marginal *R*^2^, Table 2), reflecting the effect of geographical location.

**Table 2:** Socio-economic factors associated with conservation attitudes of IOS: summary of the parsimonious ordinal mixed model

Terms of the model	*C. procera*			*L. lanceolata*		
	est. (se)	*Z*	*p*.	est. (se)	*Z*	*p*.
Age (*years old*)	−0.01 (0.57)	−0.01	0.989	0.30 (0.22)	1.35	0.174
*Young*	−1.90 (0.69)	−2.74	0.006	−0.92 (0.23)	−4.03	0.001
Residence status (*indigenous)*	2.90 (0.66)	4.41	0.001	-	-	-
Existence of market opportunities (*Yes*)	5.35 (2.42)	2.21	0.027	-	-	-
Gender (*men*)	-	-	-	−1.45 (0.30)	−4.89	0.001
Var _Village_	12.37			0.00		
Significance of village effect	0.001			0.122		

Reference levels were *Adult* for age, *Non-residents* for residence status, *No* for market opportunities, and *Women* for gender

*est.* estimates, *se* standard error, *Z Z* statistics, *p. p* value, **- =** non-significant terms

Only terms of significant factors are shown

### Factors affecting farmers’ ability to identify plus trees of IOS

**Fig. 7:**
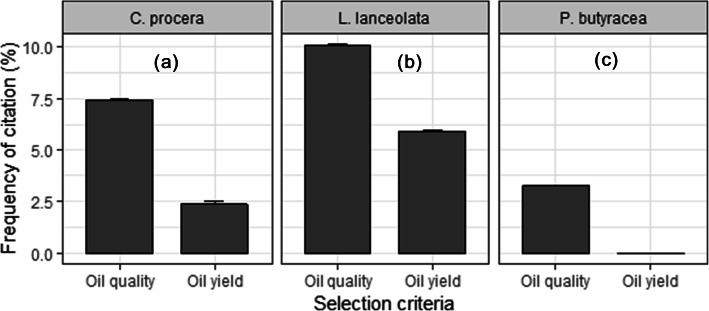
Variation of selection criteria of plus trees among the three IOS

Identification of plus trees among the three IOS was based on two criteria namely “high oil yield” and “good oil quality,” which were determined by oil extraction experience. Overall, “good oil quality” was the predominant criterion for the selection of plus trees, irrespective of the IOS (Fig. 7). Most people cited the two criteria for the selection of *L. lanceolata* followed by *C. procera*. Trees of *P. butyracea* were less cited for plus trees selection (Fig. 7). Good oil quality for *C. procera* (mainly used for medicinal purposes) was based on the oil bitterness because this was thought to determine medicinal efficacy. Good oil quality for *L. lanceolata* and *P. butyracea* (mainly used for food purposes) was based on the oil color and taste.

The selection of plus trees based on high oil yield was significantly and positively associated with conservation attitudes for *C. procera*, indicating that the better the conservation attitude, the higher the likelihood for the informant to select plus trees based on high oil yield (Table 3). The other two species did not show any significant relationship with the socio-economic factors with regard to this criterion (Table 3). However, the selection of plus trees for *C. procera* based on good oil quality was significantly associated with age, gender, actual UV, and residence status. Men, adults, elderly, and indigenous informants were more likely to select plus trees based on good oil quality than women, young, and non-resident informants. There was a positive association between actual UV and selection of plus trees for *C. procera*, indicating that the higher the actual UV (i.e., the preference), the higher the ability to select plus trees based on good oil quality. For *L. lanceolata*, significant factors were age, gender, and actual UV. Although a similar trend was observed for gender and actual UV as for *C. procera*, old informants mostly selected plus trees of *L. lanceolata* compared to adult and young informants, who showed statistically similar patterns (Table 3). For *P. butyracea*, age, residence status, and actual UV showed a significant relationship with the selection of plus trees based on good oil quality. Whereas old and adult informants showed a similar trend for selection of plus trees, young informants exhibited the lowest ability to select the trees based on the oil quality criterion. Indigenous informants had a better ability to select plus trees of *P. butyracea* than non-residents, and the higher the preference for *P. butyracea*, the higher was the ability to select its plus trees based on good oil quality (Table 3). Comparable values of conditional and marginal *R*^2^ for all IOS and selection criteria indicated that there was no or negligible variation across villages i.e., no or negligible variation across geographical locations, irrespective of the IOS (Table 3).

**Table 3:** Socio-economic factors associated with informants’ ability to identify plus trees of IOS: summary of the parsimonious binomial model

	*C. procera*			*L. lanceolata*			*P. butyracea*		
	est. (se)	*Z*	*p*.	est. (se)	*Z*	*p*.	est. (se)	*Z*	*p*.
***High productive tree***									
Intercept	−6.59 (1.27)	−5.16	0.001	-	-	-	-	-	-
Conservations attitudes	1.80 (0.45)	4.03	0.001	-	-	-	-	-	-
Var._Village_	0.00			-			-		
*R*^2^_Marginal_	45.03			-			-		
*R*^2^_Conditional_	45.03			-			-		
***Good oil quality tree***									
Intercept	−6.98 (1.27)	−5.51	0.001	−8.61 (1.32)	−6.51	0.001	−3.31 (0.54)	−6.15	0.001
Gender (*men*)	2.40 (0.82)	2.94	0.003	2.79 (0.61)	4.55	0.001	-	-	-
Age (*years old*)	0.40 (0.65)	0.61	0.542	3.16 (0.70)	4.77	0.001	0.74 (0.19)	0.04	0.969
*Young*	−1.36 (0.65)	−2.10	0.036	−2.55 (0.52)	0.00	0.989	−3.03 (0.18)	−1.65	0.001
Resident status (*indigenous*)	4.07 (0.94)	4.30	0.001	-	-	-	4.95 (1.79)	2.76	0.006
Actual use value	1.18 (0.38)	3.11	0.009	3.25 (0.62)	5.23	0.001	5.28 (0.56)	9.48	0.001
Var._Village_	1.29			1.99			0.00		
*R*^2^_Marginal_	41.05			97.27			99.92		
*R*^2^_Conditional_	57.66			98.29			99.92		

Reference levels were *Adult* for age, *Non-residents* for residence status, and *Women* for gender

*est.* estimates, *se* standard error, *Z Z* statistics, *p*. *p* value, - = non-significant terms

Only terms of significant factors are shown

## Discussion

This study assessed the preferences, conservation attitudes, and ability to select plus trees based on seeds’ traits of three IOS, and the relationship of these variables with informants’ socio-economic factors (age, gender, residence status, ethnic group, and existence of market opportunities in their village). We found differences in preferences, conservation attitudes, and plus tree selection across the three IOS. We also found species-specific patterns regarding socio-economic factors associated with IOS preferences, conservation attitudes, and selection of their plus trees.

### Preferences, conservation attitudes, and tree selection: differences among the three IOS

A preference for *C. procera* followed by *L. lanceolata* and *P. butyracea* has also been reported in previous studies that focused on their UV [5, 43]. Actually, the most well-known and cited species are those that are most often used and prioritized by communities [56]. The preference for *C. procera* suggests that it probably has a better potential to improve the livelihoods of local communities than the other two IOS. We found a positive correlation between preferences and conservation attitudes for *C. procera*. This was consistent with our prediction that species with higher actual UV receive better conservation actions from users [26] in order to ensure their long-term usage and benefits. However, this expectation was not confirmed for *L. lanceolata* because its conservation attitude was not significantly correlated with UV. Furthermore, *L. lanceolata* despite having lower UV (0.44 ± 0.03 vs. 0.61 ± 0.06), received slightly better conservation attitudes than *C. procera*. The reason could be linked to the differences in the uses of the species by local people. Indeed, oil from *C. procera* seeds is mainly used for medicinal purposes whereas that from *L. lanceolata* is used for human food [5, 57]. Assuming that food species are often prioritized because food is one of the most important basic needs, it is likely that *C. procera* receives fewer conservation actions compared to *L. lanceolata*. These results suggest that the willingness for better management of a species is primarily linked to species UV and its contribution to people livelihoods. Nonetheless, only a small proportion of informants actually practiced a conservation action toward these two species, and no conservation effort was reported for *P. butyracea*. *Lophira lanceolata* is a savanna species, contrary to the other two IOS which are gallery forest tree species. Because savannas are continuously cleared for farming and *L. lanceolata* is not listed as a priority species for special protection measures (Law No. 003-2011/AN of Article 44 on the Forest Code in Burkina Faso), trees of these species are exposed to cutting during land clearing in many places [58], making it more vulnerable [14–16]. The fact that cultivation was not practiced for any of the species might be linked to lack of local support for these species planting, and to some extent the lack of suitable management skills of these indigenous species, because most efforts were placed on exotic species [19].

Local knowledge and preferences provide information that is useful for domestication programs. In particular, an understanding of whether local people are already selecting materials among indigenous trees and the criteria locally used in selecting them have an added value for the process. Our results suggest that irrespective of the IOS, oil quality was the primary local criterion for plus tree identification, followed by the oil yield at extraction. Quality is an important factor in both commercialization and household self-consumption of oil [59, 60]. The preference based on oil quality can be explained by the fact that oils of the studied IOS are commonly used in cosmetics and human food and often in association with shea butter. For example, shea butter is often associated with *C. procera* oil in cosmetic treatments, whereas shea butter is associated with the butter of *P. butyracea* for human food consumption. For *C. procera* oil, quality is associated with oil bitterness (the more bitter, the better), and color (high clarity is desirable). Oil that is not of good quality can be linked to inadequate methods of seed storage and the processes of oil extraction, which are tightly linked to experience in the field. This is why oil extraction is commonly practiced by older informants than younger ones. This is consistent with observations by Gueye et al. [11] in Senegal and Rwanda, where older women had more practice and better knowledge of *C. procera* seed oil extraction than youngster. For *L. lanceolata* and *P. butyracea*, quality is mostly associated with taste which is expected due to their food uses.

### Socio-demographic factors associated with oil plant species preferences, conservation attitudes, and selection of “plus trees”

Although a species-specific pattern was observed, our results support that informants’ age, gender, ethnic group, residence status, and geographical location (village) are important factors in understanding IOS preferences. Similar results were observed for conservation attitudes and the ability of informants to select plus trees based on seed traits.

Consistent with our prediction of a positive relationship between preferences and gender, women had a higher UV for *C. procera* than men. Similar results were reported for the shea butter, *V. paradoxa*, another multipurpose IOS in Sub-Saharan Africa [3]. The gender-biased preference is often explained by the fact that men and women do not generally use forest resources in the same way [61]. Women are more specialized in the collection of nuts and seeds, and keener to provide the household with non-timber forest products [3]. As a result, women are expected to be more prone to the conservation of forest resources than men [62, 63]. Our results contrasted with this expectation for *C. procera* in Kénédougou province where men and women were equally involved in its conservation. However, we found a positive correlation between conservation attitudes and the existence of market opportunities for *C. procera*. This implies that the provision of market opportunities can lead to positive attitudes of local people toward the management of natural resources, as reported by N’Danikou et al. [30] in Benin for *Vitex doniana* and Akinnifesi et al. [64] in southern Africa for Miombo indigenous fruit trees. Provision of market opportunities has also been suggested as a key action for the sustainable management of trees, tree genetic resources, and the livelihoods of rural communities [65]. The economic incentives resulting from the rising demand for the oil of *C. procera* have brought local people to implement some preservation actions including ANR and the banning of tree cutting. An additional reason for this positive conservation attitude might be linked to the sharp decline of gallery forest areas [66], which has resulted in a regression of the species natural populations during recent years. Therefore, to protect their environment and fight against the shrinkage of the stream bed, rural communities are becoming more involved in species management actions. Sustainable management of wild species is a participation-driven process in which different forest resources users and stakeholders are involved. Once the priority species have been identified, another important step is to select individual trees that meet the criteria sought by local communities, because they are the central stakeholders. In the case of *C. procera*, informants orient their selection toward trees that give good oil quality and to a lesser extent high oil yield at extraction. As predicted, we found that people with better conservation attitudes were more likely to identify plus trees, but based on high oil productivity rather than good oil quality. Similarly, the higher the actual UV, the more likely the informant was to select trees based on good oil quality. Surprisingly, we did not find evidence that women were more likely to identify plus trees compared to men, but our results support the prediction of a positive relationship of age and residence status with aptitude to select plus trees. The result that men were more likely than women to identify good oil quality tree can be explained by the fact that in Kénédougou province and elsewhere in Burkina Faso especially for the Toussian ethnic group, (i) men often consume the nut of *C. procera* as a bitter kola when drinking “Dodo,” a fermented drink based on cereals (e.g., sorghum), and (ii) men often use the nuts of *C. procera* for magico-religious purposes in traditional medicine [43, 57]. These common uses are likely to make the men very familiar with the quality of the species nuts and its oil. However, the findings that men, adult and old, and indigenous informants were more likely than women, young, and non-resident informants to identify good oil quality trees suggests that these social categories are of high importance for the selection of germplasm for breeding or genetic improvement of *C. procera* in Kénédougou province. Our results for *C. procera* did not support either the hypothesis of a positive relationship of informant age and residence status with the species UV, or the expectation of differences among ethnic groups (final model after variable selection, see Table 1). This might be partly attributed to the fact that the species is relatively widespread and familiar to communities such that everyone knows and uses it [67]. Among the three IOS, *C. procera* was actually the most common gallery forest species in the area and the most valorized. This absence of a difference among ethnic groups might also be linked to the fact that we did not look at specific uses for which qualitative differences among ethnic groups can arise, or that the effect of ethnic group was confounded with another factor (e.g., village) as the univariate test showed significant variation among ethnic groups (see Fig. 4).

Similar to *C. procera*, *P. butyracea* is a specie that grows naturally in gallery forests of semi-arid areas and is subject to the same threats—shrinking of natural habitats resulting from the expansion of agriculture land—and has a relatively restricted distribution compared to *C. procera* [4, 6, 14]. However, contrary to *C. procera*, our results for *P. butyracea* support the prediction of a positive relationship of IOS UV and resident status: indigenous informants had higher UV compared to non-residents. Many parts of the species are exploited for medicinal purposes, especially by indigenous people. The reason why indigenous informants had higher actual UV can be explained by the relatively limited access to habitats of the species in Kénédougou province. Indeed, gallery forests consist of dense vegetation and sometimes shelter ritual sites with access restricted to local “Dozo” healers and hunters, native to the villages [68]. These indigenous people should have an interest in conserving the resources, but this was not the case for *P. butyracea* in Kénédougou. This might be linked to the scarcity of the species [4] in addition to the lack of marketing opportunities for its products in Kénédougou province. This lack of market opportunities might partly explain why this factor was not significant for any of the three response variables (preferences, conservation attitudes, and aptitude to select plus trees) for this species. Consistent with our prediction, we also found a positive relationship of ability to identify plus trees of *P. butyracea* with the informants’ UV of the species, residence status, and age as found for *C. procera* but with a different intensity (see the coefficients, Table 3). The indigenous community mostly used the criterion of oil quality in selecting trees for seed collection [68]. This knowledge was certainly acquired from their ancestors who used local oils for traditional pharmacopeia, food, and cultural rites. No effect of gender was detected for informants’ ability to select plus trees based on the criterion of good oil quality, suggesting that both men and women contributed equally in identifying trees producing good oil quality. The lack of differences between men and women regarding this ability might be linked to the fact that the species is less used (lower UV) and that women are not specialized in the production of this species’ seed oil.

Our predictions of positive relationships between informants’ preference and their gender, age, and resident status and differences among ethnic groups proved true for *L. lanceolata* (see Fig. 4), although the effect of residence status became insignificant when including multiple variables in the model (see Table 1). Our predictions also proved true for conservation attitudes (see Fig. 6), although only gender and age were finally retained after simplification of the model including all variables. The finding that old informants and women had higher actual UV is likely linked to the fact that knowledge accumulation is a time-related process and to division of labor, respectively, but also sociological contexts as observed for *C. procera*. Young people are also increasingly less interested in traditions, including uses of local resources due to exposure to western practices [69] which in addition to the time-dependent accumulation of knowledge on species uses, may also explain the low UV for young people. The division of labor and sociological context probably led to higher knowledge and specialization of old women for *L. lanceolata* oil extraction and uses, which resulted in higher preferences than men, as we predicted. Similar findings have been reported for the shea butter tree, *V. paradoxa* [38]. This preference probably explains why old women have better conservation attitudes toward this species than men. Contrary to the case of *C. procera*, our results did not support the prediction that the existence of market opportunities would imply better conservation attitudes. This is probably because such opportunities and seed transformation are not yet well developed for this species compared to *C. procera*. Similar to *C. procera*, we found that men had a higher likelihood than women in identifying plus trees despite not being particularly involved in their oil extraction. In Kénédougou province, men are the owners of land, as also reported for example in a study in Benin by Dadjo et al. [70]. Those who have trees of *L. lanceolata* on their land linked the oil quality of these trees to the presence of ants. According to them, trees infested by ants will produce a better oil quality. According to their belief, ants are attracted by “good things.” This may explain why men had higher aptitude than women in identifying “plus trees” based on oil quality. Ant-plant protective mutualism is a well-known relationship with many benefits for plants in community ecology [71]. However, whether this protective mutualism results in better oil quality for the protected plants remains a question that requires further investigation. Consistent with our predictions, there was also a positive relationship of aptitude to identify plus trees in *L. lanceolata* and informants’ age, residence status, and preferences for the species. Therefore, like for *C. procera*, adults and old people in addition to indigenous people can significantly contribute to the selection of interesting material for a domestication program of *L. lanceolata*.

### Implications for the sustainable management of the three IOS

*Carapa procera* was the most well-known species with the highest UV indicating this species to have a higher potential for improving the livelihoods of local people. Not all factors examined appear to be relevant for all species. Therefore, management actions should also be species-specific. Although a particular focus on women, adults, old persons, and indigenous people is relevant when designing the management of some species (e.g., *C. procera* and *L. lanceolata*), it may not be relevant for others (e.g., *P. butyracea*). The fact that no conservation practice was recorded for *P. butyracea*, whereas *L. lanceolata* received relatively better conservation attitudes than *C. procera* is suggesting that management actions can take advantage of existing practices on these two species, and improve them for better delivery. In contrast, for *P. butyracea*, higher attention is needed to ensure that the species is well conserved. The findings that adult and old informants showed a similar and better conservation attitude for *C. procera* than young informants suggest that those categories would be good stakeholders in planning conservation and sustainable management actions for this species. We found a positive association between the existence of market opportunities and better conservation attitudes for *C. procera* oil. Therefore, creating market opportunities can bring local people to adopt better management practices that will ultimately ensure the sustainable management of indigenous resources. Nevertheless, a multi-platform approach combining local people, NGOs, for profit organizations, and national offices of forest resources management is needed to ensure that the exploitation of resources is sustainable.

The conservation actions reported include protection in agroforestry parks, ANR, and tree planting. These actions have been successfully implemented for some indigenous plants like *V. paradoxa*, *Parkia biglobosa* (Jacq.) R. Br. ex G. Don, *Lannea microcarpa* Engl. & K. Krause, *Sclerocarya birrea* (A. Rich.) Hochst., *Piliostigma reticulatum* (DC.) Hochst., and *P. butyracea* Sabine [23–25]. For *V. paradoxa*, protection in agroforestry parks has improved fruit production [72]. A rotational harvest can also be taught to rural communities. For this measure to be effective, it will be necessary to promote the diversification of activities other than oil extraction. This may require further capacity building of local communities, and providing them with some facilities (e.g., equipment). For instance, in the Akonolinga locality of Cameroon, the need for domestication of *Ricinodendron heudelotii* (Baill.) Pierre ex Heckel was not a priority for rural communities until the acquisition specialized nut crushing machine. In Morocco, the same observation was made for *A. spinosa*, where the modernization of its oil extraction favors its domestication. In order to implement domestication programs, it is necessary to consider interesting traits of trees identified by rural communities with regard to good oil quality. For all three IOS, key stakeholders who will guide this process should be both adult and old people, irrespective of their gender. Thereby, we suggest participatory approaches that integrate these key informants for successful sustainable management programs of these species.

## Conclusion

This study provides evidence that the preferences, attitudes for resource conservation, and abilities of Kénédougou rural communities to select plus trees for oil-seed exploitation are species-specific. The relevance of factors such as age, gender, residence status, existence of commercialization opportunities, and geographical location (village) depended not only the species but also the variables of interest (here preferences, attitudes for resource conservation, and abilities to select plus trees for oil-seed extraction). *Carapa procera* was preferred compared to the other two species. We also demonstrated that the actual UV was associated with the ability to identify and select plus trees for oil-seed exploitation for *C. procera*. Promoting and better structuring the value chain of this species through economic and financial incentives can significantly improve livelihoods of local people while conserving its natural populations. This is expected to guarantee the sustainable exploitation of the species and serve as a good example for the other two species, namely *L. lanceolata* and *P. butyracea*. Thus, if rural communities are aware of the income opportunities offered by both species, they would receive more attention with regards to their conservation. Consequently, the three IOS would be rationally and sustainably exploited for household consumption and commercial purposes.

## Data Availability

The datasets used and/or analyzed in the current study are available from the corresponding author on reasonable request.
