# Correction to: Local preferences for three indigenous oil-seed plants and attitudes towards their conservation in the Kénédougou province of Burkina Faso, West-Africa

**DOI:** 10.1186/s13002-020-00399-9

**Published:** 2020-08-26

**Authors:** Fanta Reine Sheirita Tiétiambou, Kolawolé Valère Salako, Jésukpégo Roméo Tohoun, Amadé Ouédraogo

**Affiliations:** 1 Centre Universitaire de Gaoua, Université Nazi BONI, 01 BP 1091, Bobo-Dioulasso, 01 Burkina Faso; 2 Laboratoire de Biologie et Ecologie Végétales, Université Joseph KI-ZERBO, 03 BP 7021, Ouagadougou, 03 Burkina Faso; 3 Laboratoire de Biomathématiques et d’Estimations Forestières, Faculté des Sciences Agronomiques, Université d’Abomey-Calavi, 04 BP, 1525, Cotonou, Bénin; 4 Evolution Biologique et Ecologie, Faculté des Sciences, Université Libre de Bruxelles, CP160/12, Av. F. D. Roosevelt 50, BE-1050 Bruxelles, Belgique

**Correction to: J Ethnobiology Ethnomedicine 16, 43 (2020)**

**https://doi.org/10.1186/s13002-020-00393-1**

Following publication of the original article [1], the authors reported errors in Figs. 2, 4 and 6.

The errors were the following:

**Fig. 2:**
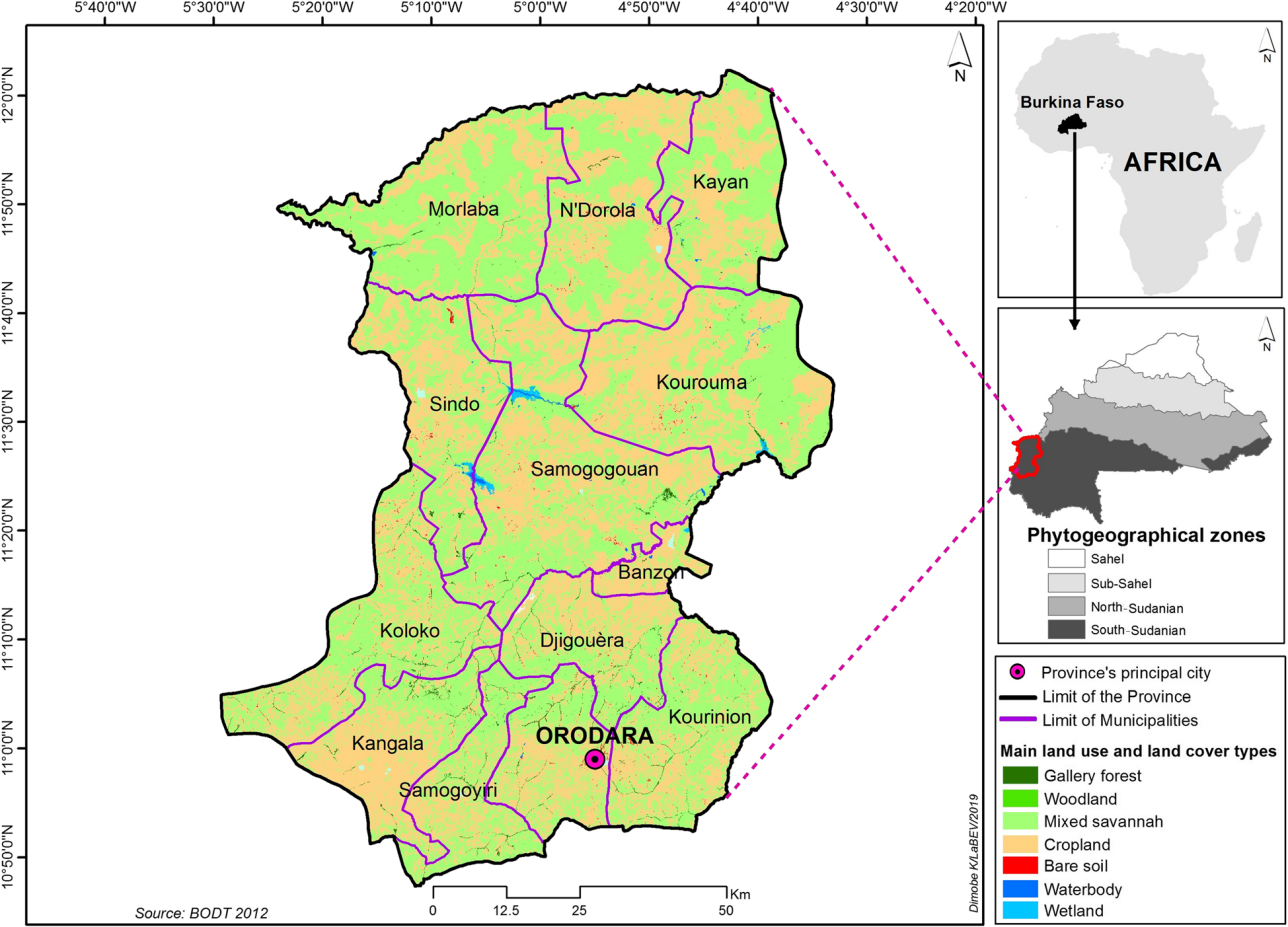
.

In the legend on ‘Phytogeographical zones’, it said ‘Southern Sudanian’ and ‘Norther Sudanian’ in place of ‘South-Sudanian’ and ‘North-Sudanian’.

**Fig. 4:**
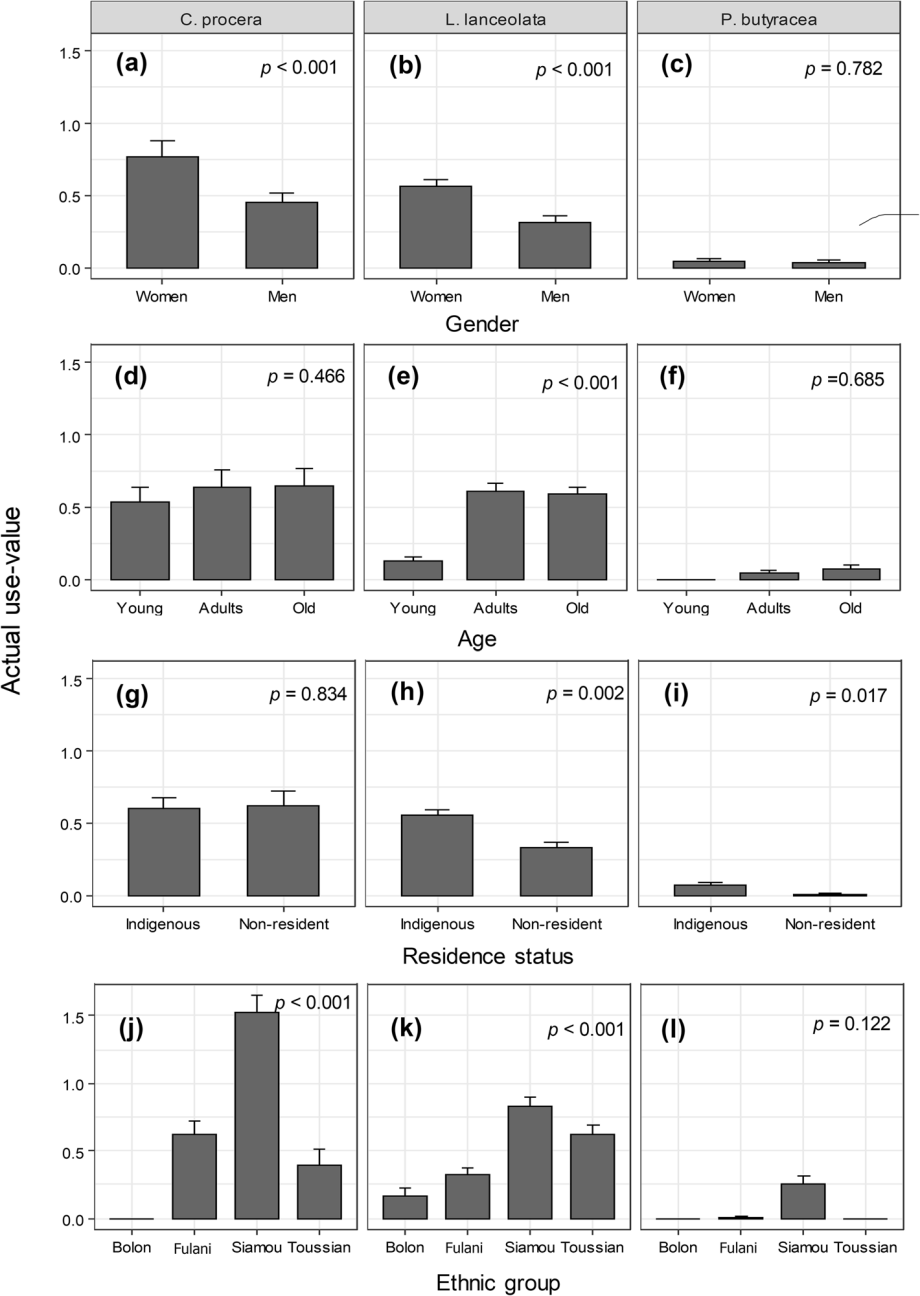
.

In panels j, k and l, it said ‘Peulh’ on the x-axis in place of ‘Fulani’.

**Fig. 6:**
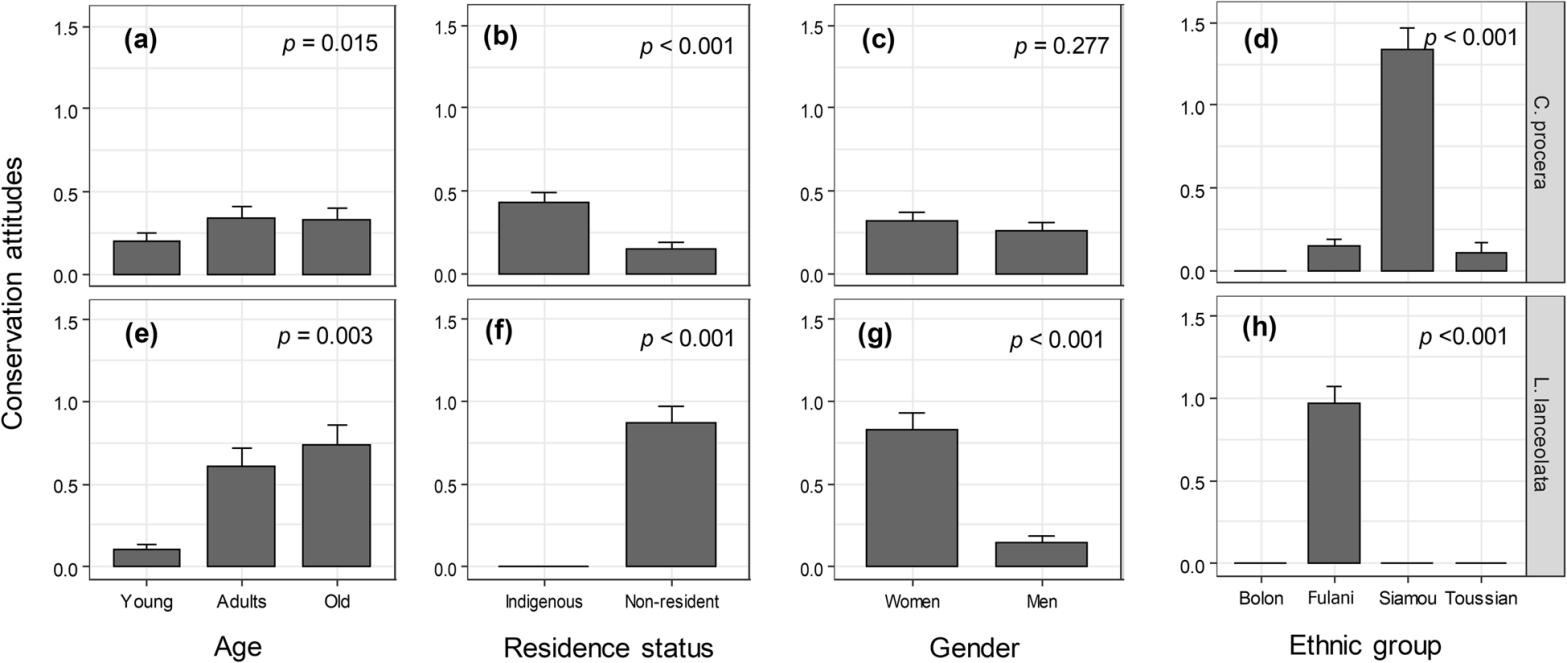
.

In panel h, it said ‘Peulh’ on the x-axis in place of ‘Fulani’.

The original article [1] has been updated to correct these errors and the (corrected) figures are included in this correction for reference.

The authors apologize for any inconvenience caused.
